# Medical subdomain classification of clinical notes using a machine learning-based natural language processing approach

**DOI:** 10.1186/s12911-017-0556-8

**Published:** 2017-12-01

**Authors:** Wei-Hung Weng, Kavishwar B. Wagholikar, Alexa T. McCray, Peter Szolovits, Henry C. Chueh

**Affiliations:** 1 Department of Biomedical Informatics, Harvard Medical School, 10 Shattuck Street, 4th Floor, Boston, MA 02115 USA; 2 Laboratory of Computer Science, Massachusetts General Hospital, 50 Staniford Street, Suite 750, Boston, MA 02114 USA; 3 Computer Science and Artificial Intelligence Laboratory, Massachusetts Institute of Technology, 32 Vassar Street, Cambridge, MA 02139 USA; 4 Department of Medicine, Massachusetts General Hospital, 55 Fruit St, Boston, MA 02114 USA

**Keywords:** Medical Decision Making, Computer-assisted, Natural Language Processing, Unified Medical Language System, Machine Learning, Deep Learning, Distributed Representation

## Abstract

**Background:**

The medical subdomain of a clinical note, such as cardiology or neurology, is useful content-derived metadata for developing machine learning downstream applications. To classify the medical subdomain of a note accurately, we have constructed a machine learning-based natural language processing (NLP) pipeline and developed medical subdomain classifiers based on the content of the note.

**Methods:**

We constructed the pipeline using the clinical NLP system, clinical Text Analysis and Knowledge Extraction System (cTAKES), the Unified Medical Language System (UMLS) Metathesaurus, Semantic Network, and learning algorithms to extract features from two datasets — clinical notes from Integrating Data for Analysis, Anonymization, and Sharing (iDASH) data repository (*n* = 431) and Massachusetts General Hospital (MGH) (*n* = 91,237), and built medical subdomain classifiers with different combinations of data representation methods and supervised learning algorithms. We evaluated the performance of classifiers and their portability across the two datasets.

**Results:**

The convolutional recurrent neural network with neural word embeddings trained-medical subdomain classifier yielded the best performance measurement on iDASH and MGH datasets with area under receiver operating characteristic curve (AUC) of 0.975 and 0.991, and F1 scores of 0.845 and 0.870, respectively. Considering better clinical interpretability, linear support vector machine-trained medical subdomain classifier using hybrid bag-of-words and clinically relevant UMLS concepts as the feature representation, with term frequency-inverse document frequency (tf-idf)-weighting, outperformed other shallow learning classifiers on iDASH and MGH datasets with AUC of 0.957 and 0.964, and F1 scores of 0.932 and 0.934 respectively. We trained classifiers on one dataset, applied to the other dataset and yielded the threshold of F1 score of 0.7 in classifiers for half of the medical subdomains we studied.

**Conclusion:**

Our study shows that a supervised learning-based NLP approach is useful to develop medical subdomain classifiers. The deep learning algorithm with distributed word representation yields better performance yet shallow learning algorithms with the word and concept representation achieves comparable performance with better clinical interpretability. Portable classifiers may also be used across datasets from different institutions.

**Electronic supplementary material:**

The online version of this article (10.1186/s12911-017-0556-8) contains supplementary material, which is available to authorized users.

## Background

Automated document classification is an effective method that can categorize documents into predefined document-level thematic labels [1]. Clinical notes, in which the medical reports are mainly written in natural language, have been regarded as a powerful resource to solve different clinical questions by providing detailed patient conditions, the thinking process of clinical reasoning, and clinical inference, which usually cannot be obtained from the other components of the electronic health record (EHR) system (e.g., claims data or laboratory examinations). Automated document classification is generally helpful in further processing clinical documents to extract these kinds of data. As such, the massive generation of clinical notes and rapidly increasing adoption of EHR systems has caused automated document classification to become an important research field of clinical predictive analytics, to help leverage the utility of narrative clinical notes [2].

Detection of the medical subdomain of a clinical note, such as cardiology, gastroenterology and neurology, may be useful to enhance the effectiveness of clinical predictive analytics by considering specialty-associated conditions [3]. Knowing the medical subdomain helps with subsequent steps in data and knowledge extraction. Training on specialist reports and applying the subdomain models on notes written by generalists, such as general practitioners and internists, will also help identify the major problems of the patient that are being described. This can be useful not only in studying the practice and validity of clinical referral patterns, but also in helping to focus attention on the most pressing medical problem subdomain of the patient.

Early research on automated document classification utilized rule-based knowledge engineering, by manually implementing a set of expert intelligence rules [1]. More recently, machine learning algorithms such as regularized logistic regression and kernel methods [4–7], and natural language processing (NLP) techniques have been utilized to support clinical decision making through risk stratification [8, 9], disease status or progression prediction using clinical narratives. For example, researchers used machine learning and NLP to perform automated clinical document classification for adjusting intensive care risk through procedure and diagnosis identification [10], detecting heart failure criteria [11], identifying adverse drug effects [12, 13], detecting the status of autism spectrum disorder [4], asthma [14], or the activity of rheumatoid arthritis [7]. For clinical administrative tasks, some studies also adopted technologies to optimize clinical workflows and improve patient safety using automated clinical document classification [6, 15].

Recently, different data representation methods have been reported to help in classifying clinical documents, for example by using lexical features, such as bag-of-words and n-grams [10, 15], adopting topic modeling methods, for example, latent Dirichlet allocation (LDA) algorithm [16], or integrating knowledge in medical ontologies such as the Unified Medical Language System (UMLS) Metathesaurus or Medical Subject Headings (MeSH) [5, 7, 17, 18], to embed clinical knowledge in documents in machine computable information.

The state-of-the-art approach to the document classification task uses neural network models with the distributed representation method [19, 20]. Instead of handcrafted feature engineering for clinical knowledge representation, the deep neural network may learn complex data representation through the algorithm itself [21]. Hughes et al. applied convolutional neural networks (CNN) with distributed word representation to medical text classification task at a sentence-level and yielded competitive performance [22, 23]. At the document-level, computer scientists applied CNN or a variant of recurrent neural network, Long Short-Term Memory (LSTM), to learn semantic representations in documents for general sentiment analysis [24–26]. CNN has also been applied at the character-level for different text classification tasks [27].

Regarding the document-level solution for detecting medical subdomains of a clinical note, Doing-Harris et al. used the clustering algorithm, with vocabulary and semantic types for their data representation, to perform the unsupervised learning task across different note types and different document sources, and yielded good performance for identifying clinical sublanguages [28]. Kocbek et al. used support vector machine (SVM) with bag-of-phrases (UMLS concepts) to detect various disease categories to classify admissions for potential diseases [5]. However, there is no study evaluating and comparing the performance of supervised shallow and deep learning algorithms with different data representations on the medical subdomain classification problem.

With the appropriate data representation, the supervised machine learning classifier for categorizing clinical notes to detect medical subdomains can augment clinical downstream applications at the medical specialty level. For example, using the medical subdomain classifier may help us understand shared syntactic and semantic structures in notes written by specialists [29], or more clinically, redirect patients with unsolved problems to the correct medical specialty for the appropriate management.

We developed a supervised machine learning-based NLP pipeline to build medical subdomain classifiers that can categorize clinical notes into medical subdomains. Specifically, we compared the performance of various shallow and deep supervised learning classifiers using different data representations, weighting strategies, and supervised learning algorithms, and we investigated the important features of medical subdomains and the portability of classifiers across two clinical datasets. We trained classifiers on one dataset and applied the best performing classifiers directly to the other dataset. We have achieved good accuracy in classifying clinical notes into their medical subdomains.

## Methods

### Overview

**Fig. 1:**
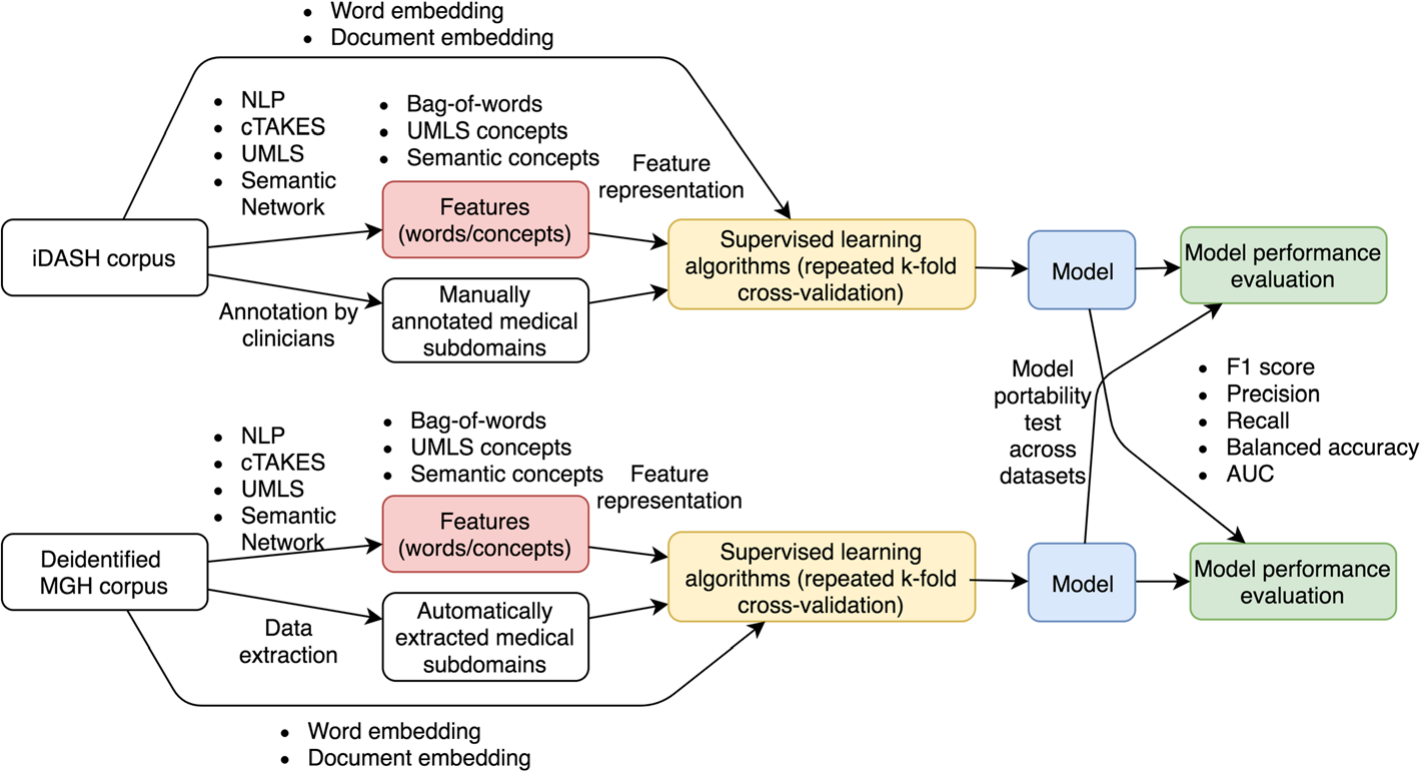
The study design. We used two datasets — clinical notes and reports from the Integrating Data for Analysis, Anonymization, and Sharing (iDASH) data repository as well as Massachusetts General Hospital (MGH) clinical notes from the Research Patient Data Registry (RPDR) data repository of the Partners HealthCare system. For each dataset, we applied and combined different data representation methods, weighting strategies, and supervised learning algorithms to build classifiers. F1 score, precision, recall, balanced accuracy and area under receiver operating characteristic curve (AUC) were used to evaluate the model performance. The model portability test across datasets was performed. We have applied the clinical NLP system, clinical Text Analysis and Knowledge Extraction System (cTAKES), the UMLS Metathesaurus, Semantic Network, and machine learning tools to construct the pipeline. The analytic pipeline has three main components, the medical concept extractor (red), model constructor (yellow), and evaluator (green)

We integrated NLP and other machine learning tools to develop our generalized clinical document classification and prediction pipeline (Fig. 1). We used two sets of clinical notes to conduct the study. The datasets were acquired from the Integrating Data for Analysis, Anonymization, and Sharing (iDASH) data repository and Massachusetts General Hospital (MGH) clinical notes in the Research Patient Data Registry (RPDR) data repository of the Partners HealthCare system [30].

### Clinical dataset

#### iDASH (integrating data for analysis, anonymization, and sharing) dataset

We downloaded 431 publicly available anonymized clinical notes or reports from the “Clinical Notes and Reports data repository” in the iDASH data repository. The iDASH data repository includes widely diverse clinical notes and reports from MedicalTranscriptionSamples.com, which is a website that collects sample notes and reports from various transcriptionists and clinical users. The iDASH documents include admission notes, discharge notes, progress notes, surgical notes, outpatient clinic notes, emergency notes, echocardiogram, CT scan, MRI, nuclear medicine, radiographs, ultrasound and radiological procedures reports. Two well-trained clinicians independently and manually annotated each document, assigning it to one of six medical subdomains: ‘Cardiology’, ‘Gastroenterology, ‘Nephrology’, ‘Neurology’, ‘Psychiatry’ and “Pulmonary disease”. Cohen’s κ coefficient of 0.97 was obtained, which represented an excellent inter-rater consistency of annotation. These annotations serve as ground truth for our learning methods.

#### MGH (Massachusetts General Hospital) dataset

The MGH dataset includes 542,744 clinical notes of 4844 patients since 2012, who had visited one of three specialist clinics (neurology, cardiology, and endocrinology) at least once in May 2016 at MGH, the tertiary care medical center in Boston, MA. We limited the note extraction query in the three specialties due to the limited data access. To allow derivation of gold standard labels of the medical subdomain for the notes without needing extensive manual annotations, we extracted all specialist-written notes and created an automated mapping script, which allows the mapping between note authors and their medical specialization using the Partners Enterprise data warehouse (EDW) physician database.

We further removed notes written by specialists with more than one specialty to ensure that each note can be classified into only one medical subdomain. After removing 386,903 notes that did not fulfill the above note selection criteria, we selected the top 24 medical subdomains among 105 medical specialties in the MGH dataset. The remaining 91,237 clinical notes were deidentified by ‘deid’ software after data filtering [31, 32], and used for the further analysis. The deidentification not only helps to protect the patients’ identities but also prevents the classification system from relying on the name of specialists for the classification task because the names are elided. The document filtering process is illustrated in Additional file 1: Figure S1. The MGH dataset was acquired through Partners Healthcare RPDR system [30], and this study was approved by the Institutional Review Board at MGH.

### Clinical word and concept representations

Appropriate clinical feature representation has been shown to improve the performance of machine learning classifiers [33]. To extract and represent interpretable clinical features, we adopted the clinical NLP annotator and parser, Apache clinical Text Analysis and Knowledge Extraction System (cTAKES) [34], and used the UMLS Metathesaurus, and Semantic Network to filter clinically relevant UMLS concepts in clinical notes [35–37].

We used the bag-of-words representation, which directly identified and normalized lexical variants from the unstructured text content, as the baseline of clinical feature representation. For clinically relevant concept identification, we selected the cTAKES analysis engine, Aggregate Plaintext UMLS Processor, to acquire UMLS concept unique identifiers (CUIs) and build feature sets. The UMLS Metathesaurus and Semantic Network were further applied to restrict the extracted UMLS CUIs within clinically relevant semantic groups and semantic types. We selected 56 semantic types within five clinically related semantic groups, which are “Anatomy (ANAT)”, “Chemicals and Drugs (CHEM)”, “Disorders (DISO)”, “Phenomena” (PHEN) and “Procedures (PROC)”. We further asked two clinicians to restrict UMLS-derived concepts from 56 to 15 semantic types (Table 1), which are most related to clinical tasks, based on clinical experiences.

**Table 1:** Fifteen semantic types selected for clinical feature representations

TUI	Semantic group	Semantic type description
T017	Anatomy	Anatomical Structure
T022	Anatomy	Body System
T023	Anatomy	Body Part, Organ, or Organ Component
T033	Disorders	Finding
T034	Phenomena	Laboratory or Test Result
T047	Disorders	Disease or Syndrome
T048	Disorders	Mental or Behavioral Dysfunction
T049	Disorders	Cell or Molecular Dysfunction
T059	Procedures	Laboratory Procedure
T060	Procedures	Diagnostic Procedure
T061	Procedures	Therapeutic or Preventive Procedure
T121	Chemicals & Drugs	Pharmacologic Substance
T122	Chemicals & Drugs	Biomedical or Dental Material
T123	Chemicals & Drugs	Biologically Active Substance
T184	Disorders	Sign or Symptom

Using clinical word and concept representations, we built features sets of (1) bag-of-words, (2) UMLS concepts, (3) UMLS concepts restricted to five semantic groups, comprising 56 semantic types, (4) UMLS concepts restricted to 15 semantic types, and also three combinations of hybrid feature sets comprising of (5) the combination of bag-of-words + UMLS concepts, (6) bag-of-words + UMLS concepts restricted to five semantic groups, comprising 56 semantic types, as well as (7) bag-of-words + UMLS concepts restricted to 15 semantic types. Through NLP, ontology and semantic filtering, clinical knowledge in clinical notes was represented in a uniform and interpretable way.

For different feature sets, we preserved all of the extracted features instead of applying additional feature selection methods to subset the features. We computed the term frequency of features as well as term frequency–inverse document frequency (tf-idf) weighting [38]. The bag-of-words features were obtained by word tokenization and word stemming using the Porter stemming algorithm [39].

### Distributed word and document representations

For the distributed document representation, we experimented with neural document embedding method, distributed memory model of paragraph vectors (PV-DM), for shallow machine learning algorithms [19]. The learned paragraph vector representations have 600-dimensions, and we used the window size of 10 words, negative sampling and frequent word subsampling rate of 10^−5^ for hyperparameter settings, and hierarchical softmax for faster training [19].

For distributed word representations, we utilized a neural word embedding model, word2vec, to process raw texts for deep learning architecture [20, 40]. As the input of deep learning classifiers, we used either the word embedding vectors trained on our input data, or the publicly available pre-trained word embedding fastText vectors [41, 42], which is a 1 million word vector trained on 16 billion tokens at the subword-level. Both the vectors have the dimensionality of 300. Words not present in the set of pre-trained words are set as a zero vector.

### Shallow learning classifiers

A total of 105 supervised shallow machine learning classifications were performed, based on 15 different data representations, the combination of seven interpretable clinical feature representations with two vector representation methods (term frequency and tf-idf weighting) as well as the paragraph vector representation, and seven supervised shallow learning algorithms. The latter included multinomial naïve Bayes (NB) algorithm as the baseline algorithm and compared against L1- or L2-regularized multinomial logistic regression, regularized SVM with linear kernel [43, 44], regularized linear SVM with stochastic gradient descent (SGD), and two ensemble algorithms, random forest and adaptive boosting. Classifiers output the class probability of all medical subdomain labels, and the label with the highest probability was regarded as the predicted result and compared against the ground truth label for evaluation.

To minimize the effect of model overfitting and model instability, repeated five-fold cross-validation was adopted in all modeling processes. Binary one-versus-rest classifiers rather than multi-class classifiers were used to reduce the evaluation complexity.

### Deep learning classifiers

The performance of neural network architectures was compared with the performance of the best-performing shallow machine learning algorithms. Two neural network architectures, CNN and convolutional recurrent neural network (CRNN) with two distributed word representations, were built based on the basic structure proposed by Kim and Shi et al. [40, 45] The CNN architecture has three sets of a one-dimensional convolutional layer with a filter size of 3 and rectified linear unit (ReLU) activation, followed by a max-pooling layer with pooling size of 2. Then a fully connected layer and a dense layer were applied for classification with a softmax function. The CRNN architecture combined CNN and bidirectional LSTM by adding the 64-cell bidirectional LSTM layer after three sets of convolution [26, 45], and before the dense layer with softmax function. We used cross entropy as the objective function and adopted the Adam optimization algorithm with the parameters provided in the original paper [46].

#### Portability test

To examine the model portability across the clinical note datasets, we applied the best feature-interpretable classifier of each dataset to classify the medical subdomains in the other dataset. In the portability test we did not consider the classifiers using distributed word or document representations due to the issue of feature interpretability.

### Evaluation

To evaluate the performance of binary classifiers, we used balanced accuracy $\left(\frac{1}{2}\times \frac{True\kern0.5em positive}{All\kern0.5em positive}\times \frac{True\kern0.5em negative}{All\kern0.5em negative}\right)$, [47], precision, recall, F1 score, and area under receiver operating characteristic curve (AUC) as performance metrics. Statistical analyses of unequal variances *t*-tests (Welch’s t-test) between groups were used as the significance test.

### Tools

The pipeline was built on cTAKES and python version 2.7.11. The Natural Language Toolkit (‘nltk’) package was used for lexical normalization (word tokenization and stemming process) of bag-of-words features generation, and for the tf-idf weighting adjustment. ‘scikit-learn’ package was selected for the supervised learning algorithms implementation and model evaluation. ‘gensim’ was used for document embeddings. ‘tensorflow’ and ‘keras’ were adopted for building deep neural networks, and neural word embeddings. Data processing, statistical analysis, and figure generation were done in Python 2.7.11 and R 3.3.2 with customized scripts. The source code of the pipeline is available online [48].

## Results

### Optimized model for medical subdomain classification

We represented the clinical features in two sets of clinical notes using different feature representation methods (Table 2).

**Table 2:** Dimension of feature sets using different data representations

Dimension of the feature set	iDASH	MGH
Bag-of-words (Vocabulary size)	8704	145,991
UMLS concepts	4751	25,457
UMLS concepts restricted to five semantic groups	4532	24,458
UMLS concepts restricted to 15 semantic types	3635	18,521
Bag-of-words + UMLS concepts	13,455	171,448
Bag-of-words + UMLS concepts restricted to five semantic groups	13,236	170,449
Bag-of-words + UMLS concepts restricted to 15 semantic types	12,339	164,512
Paragraph vector (distributed memory model)	600	600

**Fig. 2:**
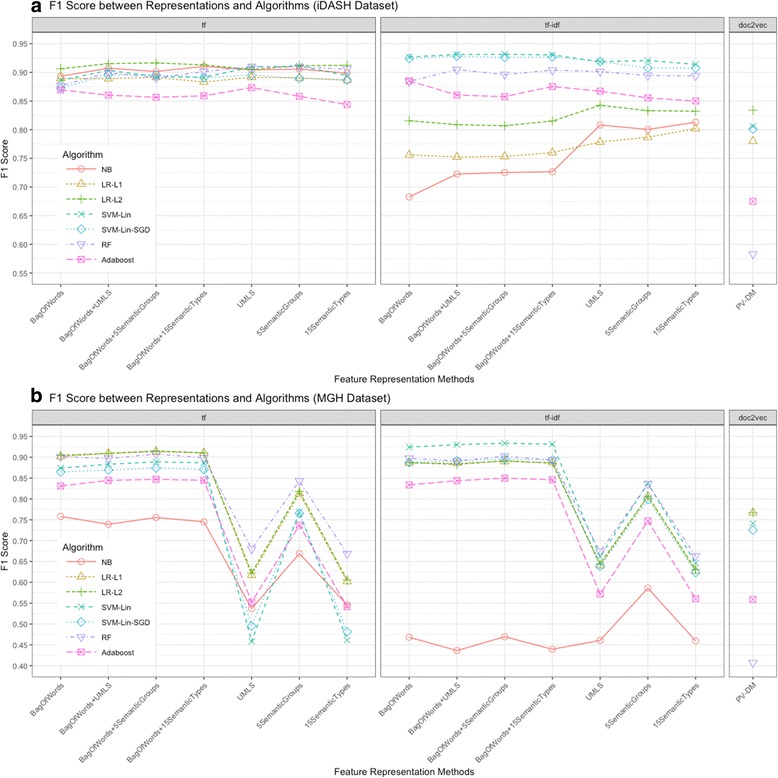
The performance of interpretable shallow learning-based classifiers (using F1 scores) built by different combinations of the clinical feature representation method with supervised learning algorithm. In both sets of clinical notes, the combination of the hybrid features of bag-of-words + UMLS concepts restricted to five semantic groups with tf-idf weighting and linear SVM yielded the optimal performance for clinical note classification based on the medical subdomain of the document. **a** F1 score of classifiers trained on iDASH dataset, **b** F1 score of classifiers trained on MGH dataset. The lines connecting data points for different clinical feature representation methods only serve to tie together the visual results from specific algorithms on different sets of features, but should not imply continuity in the horizontal axis features

We first investigated 105 combinations of data representations and supervised shallow learning algorithms to generate medical subdomain classifiers for clinical notes. The baseline classifier used the bag-of-words, term frequency representation, and NB algorithm. In the iDASH dataset, combining the hybrid features of bag-of-words + UMLS concepts restricted to five semantic groups, with tf-idf weighting and linear SVM algorithm yielded the best performing classifier for medical subdomain classification (F1 score of 0.932, AUC of 0.957), followed by bag-of-words + all UMLS concepts or using the bag-of-words + UMLS concepts restricted to 15 semantic types as the feature representation with tf-idf and linear SVM. The classifiers built by these combinations outperformed the baseline classifier with statistical significance (*p* < 0.01) (Table 3, Fig. 2 for F1 score, Additional file 1: Figure S2 for AUC).

**Table 3:** Top five best-performing interpretable shallow classifiers in iDASH and MGH datasets

Data	Feature	Vector	Algorithm	F1	AUC	*p*-value
iDASH	Bag-of-words + UMLS (5SG)	Tf-idf	SVM-Lin	0.932	0.957	<0.01
	Bag-of-words + UMLS (All)	Tf-idf	SVM-Lin	0.931	0.957	<0.01
	Bag-of-words + UMLS (15ST)	Tf-idf	SVM-Lin	0.930	0.957	<0.01
	Bag-of-words + UMLS (All)	Tf-idf	SVM-Lin-SGD	0.928	0.955	<0.01
	Bag-of-words	Tf-idf	SVM-Lin	0.927	0.955	<0.01
	**Bag-of-words**	**Tf**	**NB**	**0.893**	**0.935**	**Baseline**
MGH	Bag-of-words + UMLS (5SG)	Tf-idf	SVM-Lin	0.934	0.964	<0.01
	Bag-of-words + UMLS (15ST)	Tf-idf	SVM-Lin	0.931	0.962	<0.01
	Bag-of-words + UMLS (All)	Tf-idf	SVM-Lin	0.930	0.962	<0.01
	Bag-of-words	Tf-idf	SVM-Lin	0.924	0.958	<0.01
	Bag-of-words + UMLS (5SG)	Tf	LR-L1	0.915	0.953	<0.01
	**Bag-of-words**	**Tf**	**NB**	**0.755**	**0.867**	**Baseline**

*Abbreviation: SG* Semantic groups, *ST* Semantic types, *Tf* Term frequency, *Tf-idf* Term frequency-inverse document frequency weighting, *SVM-Lin* Linear support vector machine, *SVM-Lin-SGD* Linear support vector machine with stochastic gradient descent training, *LR-L1* L1-regularized multinomial logistic regression, *NB* Multinomial naïve Bayes. Baseline combinations are shown in bold face

In the MGH dataset, the linear SVM classifier with tf-idf weighting and the hybrid feature representation of bag-of-words + UMLS concepts restricted to five semantic groups also yielded the best performance (F1 score of 0.934, AUC of 0.964), which significantly outperformed the baseline NB classifier with the term frequency and bag-of-words combination (Table 3, Fig. 2 for F1 score, Additional file 1: Figure S2 for AUC). Relaxing the semantic feature representation also yielded optimally performing classifiers (Fig. 2). Overall, classifiers constructed by the combination of the hybrid feature representation of bag-of-words + UMLS concepts restricted to five semantic groups or 15 semantic types, with tf-idf weighting representation and linear SVM algorithms yielded better performance on classifying the clinical notes into the correct medical subdomain in both iDASH and MGH datasets.

We further examined important features by ranking coefficients of variables in the L1-regularized multinomial logistic regression classifier. The top important features of six medical subdomains in the iDASH and MGH classifiers are listed in Additional file 1: Table S2.

**Fig. 3:**
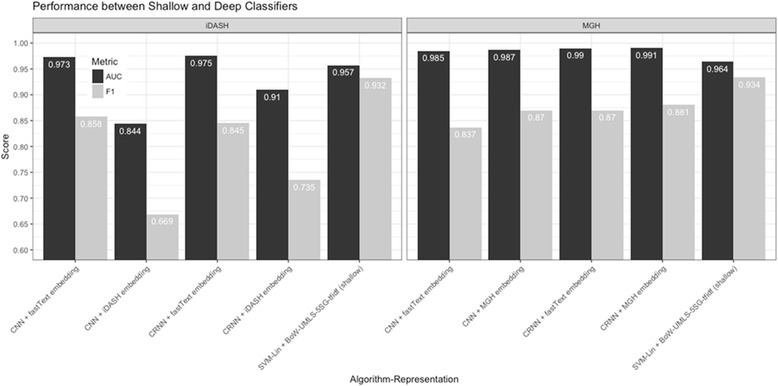
The performance comparison of different deep learning architecture and word embeddings with the best-performing shallow learning classifiers. In both datasets, the best shallow learning classifier, the combination of the hybrid features of bag-of-words + UMLS concepts restricted to five semantic groups with tf-idf weighting and linear SVM yielded the best F1 score, and comparable AUC in the medical subdomain classification task. In iDASH dataset, CNN and CRNN with pre-trained fastText word embeddings have better performance compared with using iDASH notes-trained word embedding vectors. On the contrary, CNN and CRNN with word embedding vectors trained by MGH notes yielded better performance compared with pre-trained fastText word embeddings in MGH dataset

Next, we compared the performance of the combinations of two word embedding vectors and two neural network architecture to the best classifier built by shallow learning algorithms. In the iDASH dataset, utilizing pre-trained fastText word embedding vectors with CRNN architecture yielded the comparable performing classifier for medical subdomain classification (AUC of 0.975, F1 score of 0.845), followed by fastText + CNN (AUC of 0.973, F1 score of 0.858) (Fig. 3). In the MGH dataset, using our input data for word embedding training with CRNN yielded the best performance (AUC of 0.990, F1 score of 0.881), which significantly outperformed the other classifiers, and followed by adopting fastText word embedding vectors with CRNN (Fig. 3). The deep learning architecture with distributed word representation yielded a lower F1 score in two datasets compared to the best-performing shallow learning classifier. Features in the deep learning with neural word embedding approach are not clinically interpretable due to the nature of the distributed representation.

### Error analysis

**Fig. 4:**
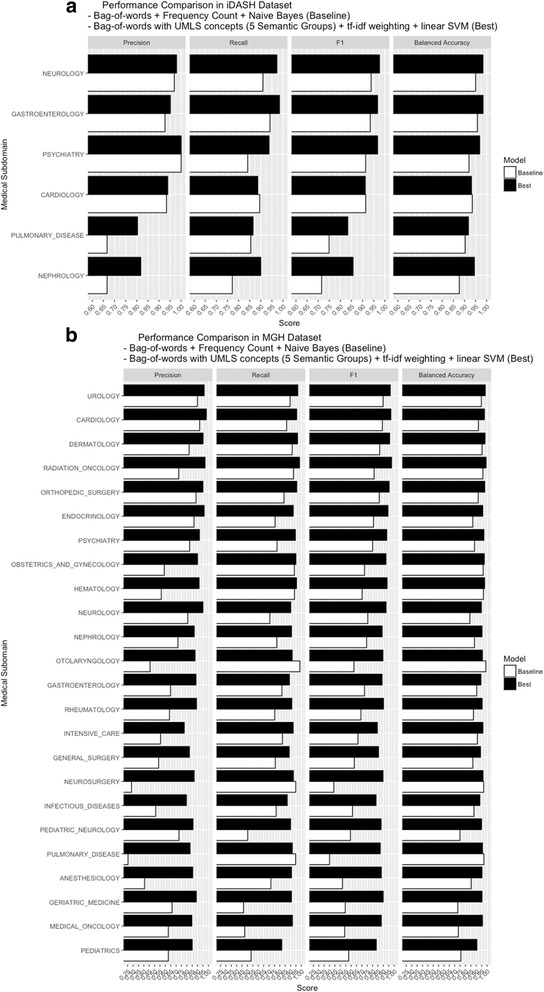
The performance across different medical subdomains in the baseline and the best interpretable classifiers on iDASH and MGH datasets. All measurements, including precision, recall, F1 score, balanced accuracy, and AUC were compared in the **a** baseline (white) and the best (black) iDASH classifiers, and the **b** baseline (white) and the best (black) MGH classifiers. Significantly improved performance is observed in the best classifier, especially in difficult to separate medical subdomains, such as ‘Anesthesiology’, “Pulmonary disease”, “Intensive care” and “Infectious diseases”

For each dataset, we compared all performance metrics between the baseline and the best-performing feature-interpretable classifiers. Balanced accuracies of the baseline and the best classifiers of iDASH dataset are 0.896 and 0.932, respectively, and balanced accuracies of the baseline and the best classifiers of MGH dataset are 0.763 and 0.925, respectively. Regardless of different combinations of the clinical feature representation and machine learning algorithm, the specificity and negative predictive value (NPV) are consistently high. However, the recall (sensitivity) and precision (positive predictive value) are low in some medical subdomains (Fig. 4).

The best-performing iDASH and MGH classifiers, which used the hybrid feature representation of bag-of-words + UMLS concepts restricted to five semantic groups, with tf-idf weighting and linear SVM, performed better compared to other classifiers. Figure 4(a) shows that the precision and F1 score of the baseline iDASH classifier are low in medical subdomains of “Pulmonary disease” (F1 score of 0.749 and precision of 0.667) and ‘Nephrology’ (F1 score of 0.715 and precision of 0.667). The recall is low in ‘Psychiatry’ (F1 score of 0.914 and recall of 0.841). In the best iDASH classifier, the F1 score and precision in the medical subdomain “Pulmonary disease” are 0.833 and 0.804, and in ‘Nephrology’ are 0.857 and 0.818, respectively. The F1 score and recall of ‘Psychiatry’ are 0.968 and 0.938, respectively. Confusion matrices of classification tasks using the baseline and the best iDASH classifiers are shown in Additional file 1: Table S3.

Figure 4(b) demonstrated that the baseline classifier for the MGH dataset yielded low precision in many medical subdomains. Nine of 24 medical subdomains have precision lower than 0.6 (‘Anesthesiology’, “General surgery”, ‘Hematology’, “Infectious diseases” “Intensive care”, ‘Neurosurgery’, “Obstetrics and gynecology”, ‘Otolaryngology’ and “Pulmonary disease”) and four of 24 medical subdomains have recall lower than 0.6 (“Geriatric medicine”, “Medical oncology”, ‘Pediatrics’ and “Pediatric neurology”). The best classifier of MGH data, however, improves most of the measurements to above 0.8, except precision of classifying the “Infectious disease” and “Intensive care” subdomains (precision of 0.797 and 0.776, respectively). F1 score of classifying all medical subdomains are above 0.83.

### Model portability

The portability of feature-interpretable classifiers built by shallow learning algorithms shows that the overall accuracy using the best iDASH classifier (with six medical subdomains) to classify medical subdomains of MGH clinical notes is 0.734. The classifier yielded the highest performance in the subdomain ‘Cardiology’ (F1 score of 0.806, precision of 0.923 and recall of 0.715), and had the lowest performance in the subdomain “Pulmonary disease” with F1 score of 0.307, precision of 0.197 and recall of 0.692. Other subdomains fall in between (Table 4).

**Table 4:** Model portability test

From iDASH to MGH					From MGH to iDASH				
Subdomain	AUC	Precision	Recall	F1	Subdomain	AUC	Precision	Recall	F1
Cardiology	0.828	0.923	0.715	0.806	Cardiology	0.731	0.829	0.500	0.624
Gastroenterology	0.802	0.396	0.691	0.503	Gastroenterology	0.832	1.000	0.664	0.798
Neurology	0.877	0.745	0.859	0.798	Neurology	0.775	0.902	0.567	0.696
Psychiatry	0.803	0.907	0.613	0.732	Psychiatry	0.941	0.794	0.900	0.844
Pulmonary	0.820	0.197	0.692	0.307	Pulmonary	0.545	1.000	0.089	0.164
Nephrology	0.770	0.573	0.561	0.567	Nephrology	0.634	0.750	0.273	0.400

The performance of using the best interpretable iDASH classifier to classify the medical subdomain of MGH clinical notes, and using the best interpretable MGH model to classify the medical subdomain of iDASH documents

The overall accuracy of using the best MGH classifier (with 24 medical subdomains) to classify medical subdomains of iDASH notes and reports is 0.520. The medical subdomain ‘Psychiatry’ had the best classification performance with F1 score of 0.844, precision of 0.794 and recall of 0.900, followed by ‘Gastroenterology’, ‘Neurology’, ‘Cardiology’, ‘Nephrology’, then “Pulmonary disease”. The overall accuracy of using the best iDASH deep learning classifier to predict MGH medical subdomain is 0.244, and the accuracy of using the best MGH deep learning classifier to predict iDASH label is 0.534.

Among top 200, 500 and 1500 features of two datasets, 6.67%, 10.93% and 16.60% of features are shared (Additional file 1: Table S4 provides the top features of each medical subdomain), respectively.

## Discussion

In this study, we found that the selection of a classifier-building combination of the data representation and supervised learning algorithm is important to yield a better-performing and portable medical subdomain classifier for clinical notes, and we show that medical subdomains can be classified accurately using the clinically interpretable supervised learning-based NLP approach. The contributions of this study include that (1) we first evaluate and compare the performance of the combinations of different data representations and supervised shallow/deep learning algorithms, including CNN and CRNN, on the medical subdomain classification using real-world unstructured clinical notes, (2) the proposed method can be a solution for building portable medical subdomain classifiers for clinical notes without medical specialization information, and (3) we have developed an open-source pipeline for future research use [48].

Regarding previous studies for medical subdomain detection in clinical documents, Doing-Harris et al. used unsupervised clustering methods with bag-of-words plus bag-of-UMLS concepts representation to cluster clinical documents and identify clinical sublanguage [28]. However, the clustering method may not yield consistent results since they are highly dependent on the initialization step. The study also only provided limited performance measurements. Kocbek et al. used the supervised solution, SVM, with the bag-of-UMLS concepts representation but focused more on disease categorization for admission notes rather than clinical subdomain classification for different note types [5]. In contrast, we tackled the medical subdomain classification by utilizing the existing information of specialty labels as the proxy of clinical subdomain and performed the supervised learning task with different shallow and deep learning algorithms. We examined the performance of using different word, concept and distributed representations as well. Similar to the finding of the sentence-level text classification task [22], our results also show that the AUCs of deep learning architecture (CNN and CRNN) with distributed word representation performs better than other top-performing shallow supervised learning algorithms, such as linear SVM and regularized multinomial logistic regression, at document classification. However, F1 scores of deep learning-based classifiers are lower than shallow classifiers. Even though shallow machine learning algorithms with clinical lexical features yielded slightly lower AUC, they can still achieve a faster and more interpretable model with reliable results and higher F1 scores, which may be practical for clinical decision making.

Among 105 classifiers with different classifier-building combinations of feature representations and shallow learning algorithms, the classifier constructed by the combination of tf-idf weighted bag-of-words + UMLS concepts restricted to specific semantic groups or semantic types as the feature representations, and linear SVM algorithm outperformed other combinations in both the iDASH and MGH clinical note datasets. For feature representation, Yetisgen-Yildiz et al. also achieved the best model performance using the word and phrase hybrid approach for clinical note classification [33]. We also adopted the similar bag-of-words and UMLS concept hybrid, which allows us to capture interpretable and important tokenized words and medical phrases that can’t be identified in concepts-only or words-only models. For example, combined features identify both the word ‘heart’ and the concept “congestive heart failure” when “congestive heart failure” appears in the text. The word ‘heart’ and the phrase concept “congestive heart failure” are both important features for a cardiology note, yet concepts-only models would identify “congestive heart failure” while words-only models would identify ‘heart’ and miss the full concept “congestive heart failure”. Using both word and concept level features can therefore maximize the utilization of information and improve clinical interpretability.

Adding UMLS concepts restricted to semantic groups or semantic types on the basis of the bag-of-words feature slightly augments the classifier performance, yet using the bag-of-words feature is necessary to yield the optimal result. Previous studies also used the feature space with both vocabulary and selected semantic concepts to cluster clinical notes with good performance [28, 49]. Semantic restriction reduces the size of the feature space by removing clinically irrelevant concepts and therefore decreases the model complexity. However, the bag-of-words feature includes some words, which may not be recognized as medical concepts by clinical NLP systems (e.g. abbreviations, neologisms), but would be important for identifying the medical subdomain of a clinical document. Therefore, combining the bag-of-words feature with semantic restricted medical concepts is useful to compensate for the disadvantages of missing those words in the pure concept approach. Many specific medical subdomains, such as ‘Psychiatry’ and ‘Neurology’, yielded good performance and portability across clinical datasets. However, some paired medical subdomains such as “Pulmonary disease” and ‘Nephrology’ are difficult to distinguish by classifiers because they often share patients with similar clinical conditions. In the iDASH classifiers, we found that the subdomains “Pulmonary disease” and ‘Nephrology’ have lower precision, and ‘Cardiology’ has relatively poor recall. This may imply that some pulmonology and nephrology cases are misclassified to cardiology. The possible cause is that patients in pulmonology and nephrology clinics may share the same features, such as dyspnea, with patients in cardiology clinics. Overlapping features lead to a harder classification task between these medical subdomains. The issue of mixed sublanguage also resulted in the limited performance in the unsupervised approach [28]. The relatively poor performance in ‘Anesthesiology’, “Infectious disease”, and “Intensive care” subdomains can also be explained by the patient similarity with other subdomains. By contrast, certain medical subdomains, for example, ‘Neurology’, “Orthopedic surgery”, ‘Psychiatry’, “Radiation oncology”, and ‘Urology’, usually yield better performance because of the uniqueness of their features.

Clinically interpretable and important features of classifiers are useful for clinicians to understand how the classifier makes its decisions. It can also be used for developing a domain ontology for NLP-driven research in specific medical domains [50]. Even though the deep learning-based approach yielded better AUCs, the interpretability of the model is still an issue, and we would suggest to use shallow models for practical use. We identified the top features of different medical subdomains in the top shallow model, but some ambiguous or clinically unrelated words and phrases also appear on the list, which indicates that the classifier fitted not only meaningful data but also noise. We also found that the important features in different datasets are both meaningful but varied. Additional file 1: Table S2 and Table S4 show that the number of overlapping features is limited. This is because the characteristics of the two sets of clinical notes are different. Notes and reports in the iDASH dataset include outpatient notes, inpatient summaries, procedure reports, and examination reports, while MGH clinical notes are mainly outpatient notes. The small overlapping of top features may also be helpful for validating our methods. The suboptimal performance of the MGH classifier portability also revealed the issue that the content of the MGH dataset is more homogeneous in comparison with the iDASH dataset. To achieve better performance of model portability, source and target data may need to have similar features.

The strength of the study is that we took advantage of the combination of clinical word and concept representations, distributed representations, and supervised shallow and deep learning algorithms for medical subdomain classification of clinical notes, which has not, to our knowledge, been explored. We used standardized terminology in the UMLS Metathesaurus for clinical feature representation, and we further identified clinically relevant UMLS concepts using semantic groups and semantic types in the Semantic Network. Using standardized terminology can be a good knowledge representation approach, which also provides the possibility of future clinical EHR system integration. We also compared the performance of word embedding vectors generated from our datasets with the publicly available pre-trained word vectors, fastText [41, 42]. The word vectors trained by our datasets may also be useful for future clinical machine learning tasks.

There are also some limitations of the study. First, we only adopted the NLP analysis tools from cTAKES. We did not examine other clinical NLP systems for performance comparison. Though cTAKES includes an NLP pipeline with promising performance [34], there are still other options, such as MetaMap from the National Library of Medicine (NLM) [51], the Clinical Language Annotation, Modeling and Processing Toolkit (CLAMP) developed by the NLP team at The University of Texas Health Science Center at Houston, and the name entity-specific tool Clinical Named Entity Recognition system (CliNER) [52]. Further investigation on different clinical NLP systems is required to understand whether cTAKES is the most suitable tool for use in predicting the medical subdomain of a clinical document. Additionally, we investigated only two clinical note datasets. To be generalizable, further investigation on more datasets is required. We also found that a few physicians’ first names appear in our feature spaces of MGH classifiers, which indicates that the process of deidentification was not perfect. Further improvement of deidentification is still required to prevent classification tasks from using the information of specific healthcare providers. For example, using deep learning to replace the current dictionary-based approach might improve performance of deidentification [53]. We also used the UMLS Metathesaurus only for concept matching, and ignored other information such as concept relationships. Searching for the possibility of increasing the interpretability of deep neural network may also further improve the performance of similar tasks. Finally, we would need to do additional external validation by experienced clinicians to integrate the medical subdomain classification into real-world clinical decision support system.

## Conclusions

Our study shows that a supervised learning-based NLP approach is useful to develop medical subdomain classifiers. The deep learning architecture with distributed word representation yields better performance, yet the shallow learning algorithm with interpretable clinical data representation has comparable results and may be more understandable and acceptable in the clinical setting. Portable classifiers may also be used across datasets from different institutions. The supervised machine learning-based NLP approach to classify the medical subdomain of a clinical note may assist clinicians to redirect patient’s unsolved problems to adequate medical specialties and experts in time purely based on the content of clinical notes. Often clinicians encounter patients’ clinical problems and dilemmas beyond their domain of expertise, which may leave questions unanswered, and result in misdiagnosis, delayed clinical care, delayed or failure to refer and even lead to inappropriate treatment and management [54]. Identifying the medical subdomain of a clinical note can also help with NLP. For example, the subdomains may generate topics, and topics may generate concepts, phrases and words via generative models for further NLP applications. We plan to integrate the information of both medical subdomains and clinical experts to build hierarchical models to improve our methods, and will adopt domain adaptation and transfer learning techniques to improve the performance of model portability to construct a generalizable solution.

## Additional file

Additional file 1: Figure S1. The Final Dataset Selection Process of MGH Dataset. **Figure S2** The performance of classifiers (using AUC) built by different combinations of the clinical feature representation method, vector representation method and supervised learning algorithm. In both datasets, the combination of the hybrid feature of bag-of-words + UMLS concepts restricted to five semantic groups with tf-idf weighting and linear SVM yielded the optimal performance for clinical note classification based on the medical subdomain of the document. (a) AUC of classifiers trained on iDASH dataset, (b) AUC of classifiers trained on MGH dataset. The lines connecting data points for different clinical feature representation methods only serve to tie together the visual results from specific algorithms on different sets of features, but should not imply continuity in the horizontal axis features. **Table S1** Representative medical subdomains in the iDASH and MGH dataset. We selected the top 24 medical subdomains from 105 medical specialties in the MGH dataset. **Table S2** Ranked top post-stemming important features (bag-of-words + UMLS concepts restricted to five semantic groups) of six medical subdomains identified by iDASH and MGH classifiers. The phrases in the parentheses are the UMLS descriptions of the corresponding UMLS CUIs. **Table S3** The confusion matrices of the classification tasks using the (a) baseline and (b) the best iDASH classifiers. **Table S4** Percentage of overlapping ranked top features of iDASH and MGH datasets (DOCX 555 kb)

## Abbreviations

AUC: Area under receiver operating characteristic curve
CLAMP: Clinical Language Annotation, Modeling and Processing Toolkit
CliNER: Clinical Named Entity Recognition system
CNN: Convolutional neural networks
CRNN: Convolutional recurrent neural network
cTAKES: Clinical Text Analysis and Knowledge Extraction System
CUI: Concept unique identifiers
EDW: Enterprise data warehouse
EHR: Electronic health record
iDASH: Integrating Data for Analysis, Anonymization, and Sharing
LDA: Latent Dirichlet allocation
LSTM: Long Short-Term Memory
MeSH: Medical Subject Headings
MGH: Massachusetts General Hospital
NB: Naïve Bayes
NLM: National Library of Medicine
NLP: Natural language processing
NPV: Negative predictive value
PV-DM: Distributed memory model of paragraph vectors
ReLU: Rectified linear unit
RPDR: Research Patient Data Registry
SGD: Stochastic gradient descent
SVM: Support vector machine
tf-idf: Term frequency-inverse document frequency
UMLS: Unified Medical Language System
