# Supplementary material for: Medical subdomain classification of clinical notes using a machine learning-based natural language processing approach
Source: BMC Med Inform Decis Mak. 2017 Dec 1;17:155. doi: 10.1186/s12911-017-0556-8 (PMC5709846; doi:10.1186/s12911-017-0556-8)
Supplement: Additional file 1: Figure S1. — The Final Dataset Selection Process of MGH Dataset. Figure S2 The performance of classifiers (using AUC) built by different combinations of the clinical feature representation method, vector representation method and supervised learning algorithm. In both datasets, the combination of the hybrid feature of bag-of-words + UMLS concepts restricted to five semantic groups with tf-idf weighting and linear SVM yielded the optimal performance for clinical note classification based on the medical subdomain of the document. (a) AUC of classifiers trained on iDASH dataset, (b) AUC of classifiers trained on MGH dataset. The lines connecting data points for different clinical feature representation methods only serve to tie together the visual results from specific algorithms on different sets of features, but should not imply continuity in the horizontal axis features. Table S1 Representative medical subdomains in the iDASH and MGH dataset. We selected the top 24 medical subdomains from 105 medical specialties in the MGH dataset. Table S2 Ranked top post-stemming important features (bag-of-words + UMLS concepts restricted to five semantic groups) of six medical subdomains identified by iDASH and MGH classifiers. The phrases in the parentheses are the UMLS descriptions of the corresponding UMLS CUIs. Table S3 The confusion matrices of the classification tasks using the (a) baseline and (b) the best iDASH classifiers. Table S4 Percentage of overlapping ranked top features of iDASH and MGH datasets (DOCX 555 kb) [file 12911_2017_556_MOESM1_ESM.docx]

**Additional File 1**


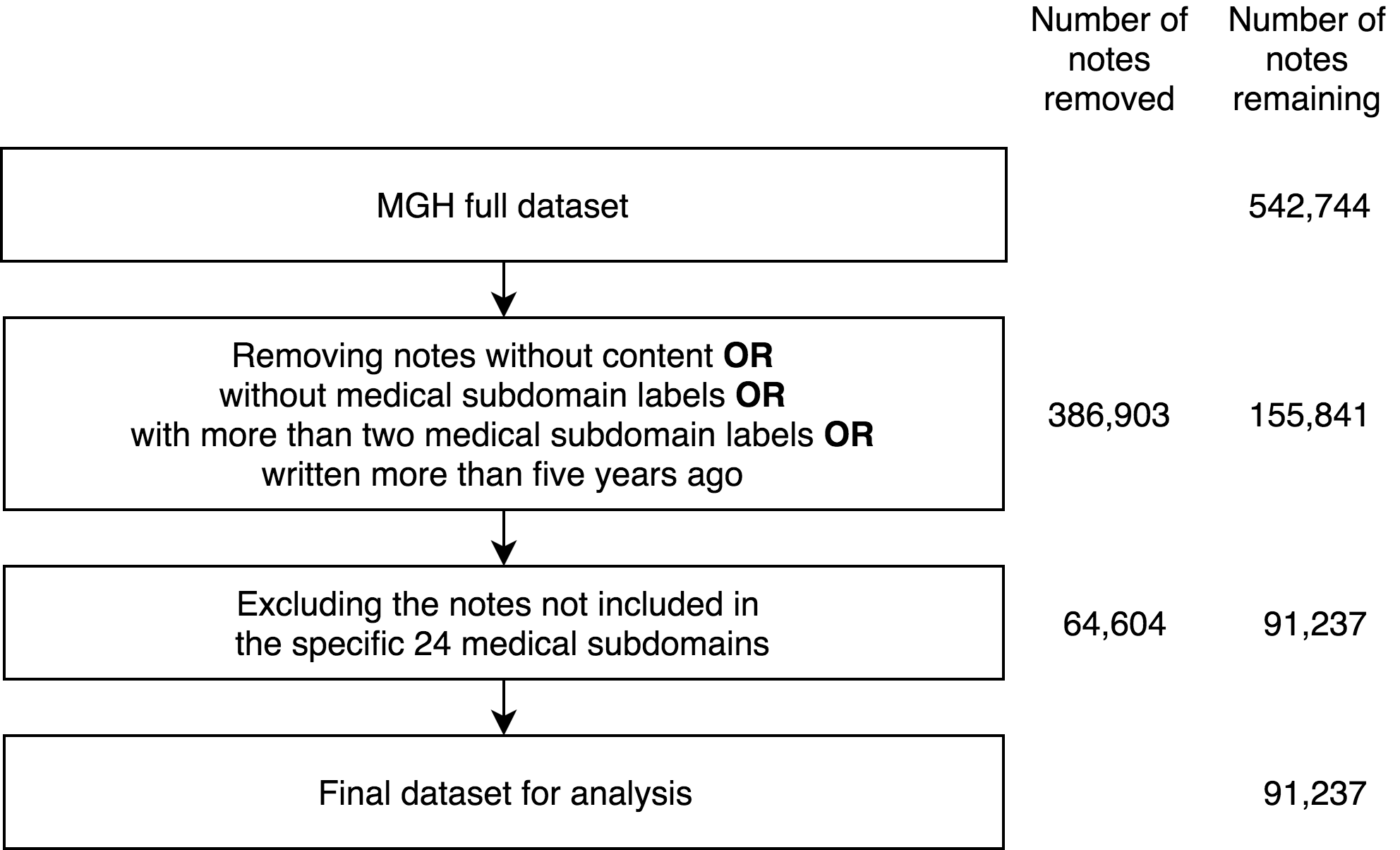


Supplementary figure 1. The Final Dataset Selection Process of MGH Dataset.


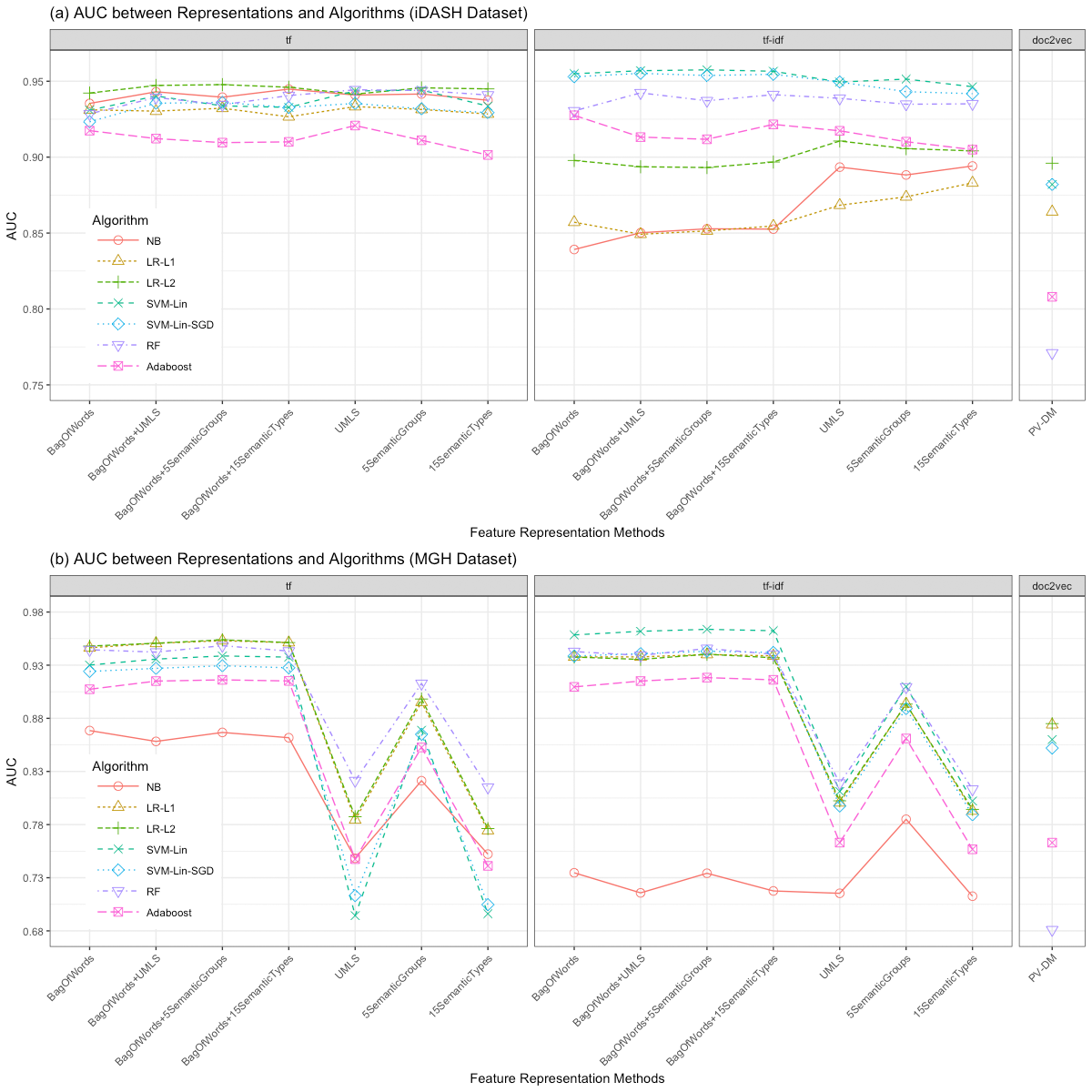


Supplementary figure 2. The performance of classifiers (using AUC) built by different combinations of the clinical feature representation method, vector representation method and supervised learning algorithm. In both datasets, the combination of the hybrid feature of bag-of-words + UMLS concepts restricted to five semantic groups with tf-idf weighting and linear SVM yielded the optimal performance for clinical note classification based on the medical subdomain of the document. (a) AUC of classifiers trained on iDASH dataset, (b) AUC of classifiers trained on MGH dataset. The lines connecting data points for different clinical feature representation methods only serve to tie together the visual results from specific algorithms on different sets of features, but should not imply continuity in the horizontal axis features.

Supplementary table 1. Representative medical subdomains in the iDASH and MGH dataset. We selected the top 24 medical subdomains from 105 medical specialties in the MGH dataset.

| Medical Subdomain | Number of Documents  (iDASH) | Number of Documents  (MGH) |
| --- | --- | --- |
| Cardiology | 116 | 20,928 |
| Endocrinology | - | 12,395 |
| Neurology | 97 | 10,974 |
| Pediatrics | - | 4,790 |
| General surgery | - | 4,388 |
| Dermatology | - | 4,067 |
| Psychiatry | 30 | 3,734 |
| Gastroenterology | 110 | 3,188 |
| Orthopedic surgery | - | 3,053 |
| Geriatric medicine | - | 2,092 |
| Urology | - | 2,090 |
| Anesthesiology | - | 1,979 |
| Nephrology | 22 | 1,936 |
| Medical oncology | - | 1,881 |
| Obstetrics and gynecology | - | 1,784 |
| Infectious diseases | - | 1,729 |
| Pediatric Neurology | - | 1,655 |
| Rheumatology | - | 1,536 |
| Otolaryngology | - | 1,473 |
| Radiation oncology | - | 1,445 |
| Neurosurgery | - | 1,414 |
| Hematology | - | 1,036 |
| Intensive care | - | 907 |
| Pulmonary disease | 56 | 763 |
| Total | 431 | 91,237 |

Supplementary table 2. Ranked top post-stemming important features (bag-of-words + UMLS concepts restricted to five semantic groups) of six medical subdomains identified by iDASH and MGH classifiers. The phrases in the parentheses are the UMLS descriptions of the corresponding UMLS CUIs.

| Top Features in iDASH Model | Top Features in MGH Model |
| --- | --- |
| CARDIOLOGY |  |
| palpit \| test \| three \| doxycyclin \| myocardi \| echocardiogram \| c0817096 (Chest) \| daili \| ischem \| deni \| use \| beat \| per \| mitral \| motion \| venou \| syncop \| pressur \| c0013516 (Echocardiography) \| c0018792 (Heart Atrium) \| minut \| bypass \| stress \| diabet \| pain \| obes \| left \| valv \| systol \| obtain \| implant \| done \| c1281570 (Entire heart) \| arteri \| stenosi \| sinu \| function \| follow \| blood \| lead \| aortic \| chest \| rate \| studi \| ventricular \| c0018787 (Heart) \| coronari \| atrial \| heart \| cardiac | diagnost \| best \| push \| midfoot \| flare \| normalwith \| seleznev \| major \| coupl \| panel \| echo \| cholesterolhdl \| cholesterolldl \| urgent \| infect \| regard \| recreat \| medrol \| lyte \| c0013146 (Drug abuse) \| disposit \| rheum \| educationcounsel \| c0400018 (Diagnostic endoscopic examination on colon) \| statu \| oht \| mph \| reevalu \| pelagia \| tereza \| jvp \| mark \| sign \| facc \| interv \| languag \| hesit \| c0438105 (Examination finding) \| beeper \| c1287400 (History finding) \| pgi \| mba \| transcrib \| preliminari \| document \| sincer \| electron \| kind \| narr \| fisher |
| GASTROENTEROLOGY |  |
| also \| portion \| c0009378 (colonoscopy) \| pancrea \| appendix \| c0000737 (Abdominal Pain) \| ulcer \| diet \| gastric \| endoscopi \| chang \| posit \| c0021853 (Intestines) \| vomit \| duodenum \| scope \| symptom \| rectum \| duct \| mucosa \| visual \| c0009368 (Colon structure (body structure)) \| advanc \| c1278925 (Entire cecum) \| c0000726 (Abdomen) \| c0007531 (Cecum) \| discuss \| rectal \| stool \| c0038351 (Stomach) \| colonoscop \| transfer \| given \| endoscop \| pelvi \| nausea \| stomach \| liver \| without \| bleed \| procedur \| lesion \| abdomen \| cecum \| esophagu \| bowel \| colonoscopi \| polyp \| colon \| abdomin | go \| c0079304 (Esophagogastroduodenoscopy) \| page \| mgday \| vaccin \| rub \| inquiri \| colizzo \| empti \| methocarbamol \| c0231377 (At risk for impaired home maintenance management) \| gallop \| happi \| c0345904 (Malignant neoplasm of liver) \| dear \| saw \| hsm \| perian \| eu \| egd \| c0557061 (Discussion (procedure)) \| relax \| harvoni \| ppi \| crohn \| c0392916 (Intracellular ferritin) \| endoscop \| phd \| ferritin \| c0014245 (Endoscopy (procedure)) \| impressionplan \| heent \| mcv \| precancer \| requisit \| thiim \| lmd \| pleasur \| rheum \| khuyen \| data \| mrn \| c0719635 (DOS brand of docusate sodium) \| c1299487 (Patient name) \| outsid \| gastroenterolog \| cmd \| ananthakrishnan \| ashwin \| tel |
| NEPHROLOGY |  |
| c0019004 (Hemodialysis) \| ultrasound \| regular \| serum \| c0203408 (Echography of kidney) \| go \| c0555903 (Total protein measurement) \| anemia \| chronic \| promis \| found \| protein \| unit \| get \| manag \| echotextur \| bladder \| control \| size \| care \| recent \| endstag \| c1278978 (Entire kidney) \| hematuria \| stage \| red \| secondari \| c0022661 (Kidney Failure, Chronic) \| cyst \| need \| hydronephrosi \| c0022658 (Kidney Diseases) \| postvoid \| transplant \| ureter \| blood \| hypertens \| hospit \| histori \| c0022646 (Kidney) \| clear \| hemodialysi \| cell \| failur \| day \| creatinin \| diseas \| urin \| kidney \| renal | c1304686 (Finding of pH) \| physiolog \| levoflox \| c2916969 (Renewal) \| c0227614 (Left kidney) \| chaga \| eat \| asymptomat \| friday \| basic \| ratg \| allograft \| c1269870 (Entire thoracic spine) \| vermont \| allow \| hill \| renal \| rise \| c0085593 (Chills) \| hotel \| nyc \| induct \| c0554756 (Doppler studies) \| midnight \| uincreas \| nuclear \| litholink \| male \| txp \| ckd \| meantim \| ext \| court \| prograf \| cellcept \| c0042014 (Urinalysis) \| golf \| mwiu \| pager \| seem \| phosphoru \| hmd \| particip \| epidur \| kayexal \| nephrolog \| kalim \| sahir \| simic |
| NEUROLOGY |  |
| episod \| matter \| sensori \| oper \| hematoma \| wave \| addit \| c0016928 (Gait) \| see \| c0228174 (Cerebral hemisphere structure (body structure))\| region \| drug \| consist \| electroencephalogram \| symmetr \| sever \| tempor \| cycl \| eeg \| difficulti \| c0013819 (Electroencephalography) \| mri \| motor \| unremark \| low \| show \| awak \| memori \| tumor \| muscl \| cerebr \| nerv \| huntington \| head \| throughout \| speech \| gait \| neurolog \| subdur \| husband \| frontal \| note \| movement \| weak \| bilater \| record \| brain \| seizur \| headach \| activ | bilki \| cranial \| professor \| neurol \| mrcp \| remaind \| funduscop \| interview \| donepezil \| adusumilli \| picc \| c0023524 (Leukoencephalopathy, Progressive Multifocal) \| recap \| cheng \| fellowship \| mask \| mestinon \| nmdmph \| righthand \| buspar \| moo \| cellcept \| afib \| jcv \| exmnd \| certifi \| fellow \| wang \| toelchemali \| onfi \| lipoprotein \| c0586177 (Report status) \| softwar \| caller \| ivig \| noncontributori \| neurolog \| zelim \| key \| jhgjjm \| josna \| copaxon \| lifethreaten \| neurooncolog \| elchemali \| wac \| phd \| neuromuscular \| msph \| zeina |
| PSYCHIATRY |  |
| attent \| ideat \| unknown \| iii \| laboratori \| c0004457 (Axis vertebra) \| abcd \| orient \| person \| seroquel \| physic \| thought \| abc \| anxieti \| unabl \| father \| hallucin \| surgeri \| want \| c0344315 (Depressed mood) \| current \| substanc \| one \| bipolar \| mother \| consult \| past \| feel \| year \| famili \| pain \| secondari \| prn \| state \| mood \| patient \| histori \| medic \| axi \| psychiatr \| mental \| abus \| problem \| behavior \| time \| treatment \| depress \| deni \| disord \| quot | geriatr \| c0565867 (delivery method) \| particip \| axi \| bring \| appreci \| experi \| atyp \| basic \| psychiatr \| write \| cancel \| mirtazapin \| genitourinari \| showreschedul \| c1170371 (Lexapro)\| end \| health \| lexapro \| memori \| seen \| script \| session \| voic \| psych \| mental \| haldol \| spirit \| copi \| card \| reschedul \| luri \| sertralin \| licsw \| show \| adult \| discontinu \| qh \| fromhal \| psychiatrist \| lithium \| neuropsycholog \| ahead \| wish \| north \| secur \| sarsha \| waterfront \| psychopharmacolog \| psychiatri |
| PULMONARY \| DISEASE |  |
| lavag \| fontan \| day \| wheez \| tube \| secret \| leftsid \| babi \| found \| effus \| ventil \| xray \| pneumothorax \| pneumon \| short \| c0010200 (Coughing) \| improv \| statu \| c0006290 (Bronchoscopy) \| obstruct \| bronchoscop \| bid \| well \| appear \| trachea \| airway \| c0024115 (Lung diseases)\| shunt \| satur \| diseas \| evalu \| capac \| right \| time \| room \| daughter \| c0024109 (Lung) \| c1278908 (Entire lung) \| pneumonia \| chest \| oxygen \| bronchoscopi \| cough \| breath \| respiratori \| upper \| predict \| lobe \| pulmonari \| lung | wors \| satur \| evalu \| ashwin \| c0024554 (Male gender) \| care \| suggest \| osa \| c1579761 (Lunesta) \| lunesta \| c0019079 (Hemoptysis) \| levaquin \| mark \| instil \| jugular \| hemoptysi \| azithromycin \| denmark \| proair \| saturday \| c0013404 (Dyspnea) \| dramat \| advis \| region \| associ \| c0582147 (At risk of infection) \| c0519671 (Immunology procedure) \| print \| yesterday \| immunolog \| excus \| fellow \| intern \| directli \| c0406810 (Carney Complex) \| medicin \| speak \| explain \| express \| director \| sputum \| name \| bactrim \| c0022688 (Natural Killer Cells) \| emd \| machin \| pulmonari \| advair \| truli \| attest |

Supplementary table 3. The confusion matrices of the classification tasks using the (a) baseline and (b) the best iDASH classifiers.

(a)

| Truth \ Predicted | Cardiology | Gastroenterology | Neurology | Psychiatry | Pulmonary | Nephrology |
| --- | --- | --- | --- | --- | --- | --- |
| Cardiology | 325 | 0 | 4 | 0 | 12 | 7 |
| Gastroenterology | 8 | 306 | 7 | 3 | 3 | 3 |
| Neurology | 0 | 0 | 282 | 6 | 3 | 0 |
| Psychiatry | 0 | 0 | 0 | 90 | 0 | 0 |
| Pulmonary | 27 | 6 | 15 | 5 | 112 | 3 |
| Nephrology | 3 | 13 | 2 | 3 | 1 | 44 |

(b)

| Truth \ Predicted | Cardiology | Gastroenterology | Neurology | Psychiatry | Pulmonary | Nephrology |
| --- | --- | --- | --- | --- | --- | --- |
| Cardiology | 327 | 1 | 2 | 0 | 15 | 3 |
| Gastroenterology | 10 | 314 | 1 | 0 | 5 | 0 |
| Neurology | 0 | 0 | 285 | 6 | 0 | 0 |
| Psychiatry | 0 | 0 | 0 | 90 | 0 | 0 |
| Pulmonary | 25 | 0 | 5 | 0 | 135 | 3 |
| Nephrology | 7 | 4 | 0 | 0 | 1 | 54 |

Supplementary table 4. Percentage of overlapping ranked top features of iDASH and MGH datasets.

| \ Subdomain  Top N features | Cardiology | Gastroenterology | Nephrology | Neurology | Psychiatry | Pulmonary |
| --- | --- | --- | --- | --- | --- | --- |
| 200 | 4 | 6 | 5 | 9.5 | 8.5 | 7 |
| 500 | 8.6 | 11.4 | 8.2 | 13.6 | 12.4 | 11.4 |
| 1500 (10%) | 15.9 | 15.6 | 15.3 | 19.1 | 16.8 | 16.9 |
